# circGPA: circRNA functional annotation based on probability-generating functions

**DOI:** 10.1186/s12859-022-04957-8

**Published:** 2022-09-27

**Authors:** Petr Ryšavý, Jiří Kléma, Michaela Dostálová Merkerová

**Affiliations:** 1 Department of Computer Science, Faculty of Electrical Engineering, Czech Technical University in Prague, Prague, Czech Republic; 2 Department of Genomics, Institute of Hematology and Blood Transfusion, Prague, Czech Republic

**Keywords:** Circular RNA, Annotation term, Interaction network

## Abstract

Recent research has already shown that circular RNAs (circRNAs) are functional in gene expression regulation and potentially related to diseases. Due to their stability, circRNAs can also be used as biomarkers for diagnosis. However, the function of most circRNAs remains unknown, and it is expensive and time-consuming to discover it through biological experiments. In this paper, we predict circRNA annotations from the knowledge of their interaction with miRNAs and subsequent miRNA–mRNA interactions. First, we construct an interaction network for a target circRNA and secondly spread the information from the network nodes with the known function to the root circRNA node. This idea itself is not new; our main contribution lies in proposing an efficient and exact deterministic procedure based on the principle of probability-generating functions to calculate the *p*-value of association test between a circRNA and an annotation term. We show that our publicly available algorithm is both more effective and efficient than the commonly used Monte-Carlo sampling approach that may suffer from difficult quantification of sampling convergence and subsequent sampling inefficiency. We experimentally demonstrate that the new approach is two orders of magnitude faster than the Monte-Carlo sampling, which makes summary annotation of large circRNA files feasible; this includes their reannotation after periodical interaction network updates, for example. We provide a summary annotation of a current circRNA database as one of our outputs. The proposed algorithm could be generalized towards other types of RNA in way that is straightforward.

## Introduction

Recent research has found circular RNAs (circRNAs) to be broadly expressed in eukaryotes in tissue- and species-specific manner [1]. Some circRNAs have already been shown to regulate gene expression and potentially relate to diseases [2–4]. Due to their stability, circRNAs can also be used as biomarkers for diagnosis [2, 5, 6]. However, the function of most circRNAs remains unknown.

Functional annotation aims to attach biological information to genomic elements. The traditional functional annotation stems from sequential similarity with annotated sequences. Statistically significant similarity often reflects common ancestry and then also a common function [7]. This reasoning applies mostly to genes and proteins. CircRNAs are known to regulate gene expression by influencing the transcription, the mRNA turnover, and translation by sponging RNA-binding proteins and microRNAs [8]. Their annotation databases often include basic information on their tissue-specificity, disease associations, and miRNA interactions [9]. Furthermore, advanced circRNA annotations could be obtained from the knowledge of their interaction with miRNAs and all other interactions of these miRNAs [10]. At the same time, the circRNA annotations may stem from the known annotations of their host genes [11].

In this paper, we propose an algorithm that allows for annotating circRNAs with annotation terms as, for example, gene-ontology terms, phenotype terms, or diseases. The algorithm implements an integrative approach which firstly constructs an interaction network for a target circRNA, and secondly, spreads the information from the network nodes with the known function to the root circRNA node. The algorithm employs the simple principle that a circRNA should be annotated with a term that is over-represented in the set of its interacting nodes. The most common solution is to quantify this over-representation in terms of *p*-value through stochastic algorithms [6, 12–15]. An analogical principle has also been implemented in protein function annotation [16]. The main disadvantage of these algorithms is their randomness and low sampling efficiency. They can be computationally intensive, especially when estimating low *p*-values in multiple testing settings frequent in genetics [17]. The low *p*-values need to be estimated accurately in order to identify and possibly sort the most interesting terms for the circRNA under examination; moreover, the *p*-values must be estimated with high accuracy to control the family-wise error rate. There have been numerous past efforts to reduce the computational burden of stochastic algorithms. Those which are universally applicable [18, 19] rely on stopping early when the *p*-value is obviously large. The efficiency of these approaches depends on the frequency of low *p*-values (truly interesting annotation terms in our case), acceleration in the order of tens is typically reported. More efficient algorithms are often dedicated to specific tasks such as mean comparison in two-sample tests where the individual runs could be partitioned with *p*-values showing a predictable trend across the partitions [17]. For example, these methods can be used for the identification of differentially expressed genes. To conclude, bulk annotation of multiple circRNAs remains computationally challenging for stochastic algorithms.

Our main contribution lies in the proposal of an efficient and exact procedure that is based on the principle of probability-generating functions [20]. For this reason, we named our algorithm circGPA (circRNA generating-polynomial annotator). The algorithm has four steps. First, an interaction network for a circRNA is constructed. Second, a statistic that quantifies the size of the neighborhood of the circRNA that is annotated with a term of certain cardinality is introduced. Third, the probability mass function of the statistic, which is a discrete random variable, is represented as a power series (the generating function). Fourth, the coefficients of the power series serve to calculate the *p*-value for the pair of circRNA and annotation term efficiently and exactly.

We show that circGPA is both more effective and efficient than the commonly used stochastic sampling approach; in particular, circGPA calculates all the *p*-values exactly, and it is at least two orders of magnitude faster than stochastic sampling. This feature enables the summary annotation of large circRNA files, including their reannotation after periodical interaction network updates, for example. In the supplement, we provide a summary annotation of a current circRNA database as one of our outputs. The annotation contains around 3000 circRNAs available in the CircInteractome database and about 10,000 annotation terms taken from the C5 category of MSigDB database (ontology gene sets consisting of the gene-ontology terms and the human phenotype ontology). To illustrate the practical significance of the new algorithm, the summary annotation was processed in 20 months of CPU time^1^, with the stochastic approach this would be technically unreachable. circGPA, together with the code used to generate the figures in this paper, is publicly available on https://github.com/petrrysavy/circgpa-paper. The outputs of our code can be downloaded from https://ida.fel.cvut.cz/~rysavy/circgpa/. The proposed algorithm could be generalized in a straightforward manner towards different types of RNA that could be characterized with interaction graphs of similar properties that we show in this paper.

The rest of our paper is organized as follows. The "Problem statement" section defines the problem to be solved as root node classification in tripartite circRNA interaction graph. The "Proposed statistic" section introduces the key interaction statistic for a paired circRNA and annotation term. The statistic is easy to compute; however, its *p*-value estimation is more time-consuming. For this reason, the "*p*-value calculation" section introduces an exact *p*-value calculation algorithm based on generating functions. The "Implementation" section gives the algorithm pseudocode as well as further details on interaction network construction. The basic concepts are summarized in an all-in-one example in the "All in one example" section. The "Results" section experimentally evaluates the new algorithm and compares it with the Monte Carlo algorithm. The "Conclusion" section concludes the paper and outlines future work.

## Materials and methods

### Problem statement

This paper aims to devise an algorithm to annotate circRNA molecules with annotation terms. To do so, we exploit the interaction graph between circRNA–miRNA and miRNA–mRNA molecules. As we assume that the annotations of circRNAs are independent, we can process the individual circRNAs sequentially and restrict ourselves to a single circRNA molecule in our description. Assume a fixed ordering on miRNA and mRNA molecules. Assume that the count of mRNAs (miRNAs) is $|m|$ ($|\mu |$).

**Fig. 1:**
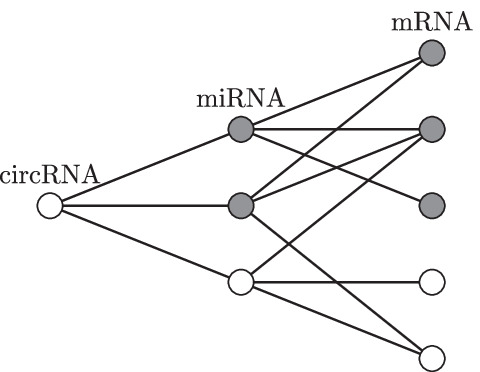
An example of a network. The grey nodes are part of the annotation term. The circRNA of interest interacts with all three miRNAs, out of which two are annotated with the term of interest. There are five mRNAs, three of them annotated with the term. In the graph, we might find three paths from the circRNA to a miRNA and nine paths from the circRNA to a mRNA. Out of those, $2+6$ terminate in an annotated mi/mRNA, resulting in $s(c, g) = 8$

Formally, we can define the interaction graph between the selected circRNA and miRNAs using a vector $\mathbf {a}^{\mu , c}\in \{0,1\}^{|\mu |}$ where each field represents whether a particular miRNA interacts with the circRNA. Interactions between miRNAs and mRNAs are represented by an adjacency matrix $\mathbf {A}^{m, \mu }\in \{0,1\}^{|m|\times |\mu |}$ where each row is a vector indicating which miRNAs interact with a particular mRNA.^2^ We assume that the graph edges are directed only from circRNA to miRNA and from miRNA to mRNA, so that a directed path cannot connect two molecules of the same type. A simple network is shown in Fig. 1.

In our notation, an annotation term will be defined by a set of mRNAs and miRNAs it annotates. The membership of mRNAs (miRNAs respectively) is formalized using binary vectors $\mathbf {g}^m\in \{0,1\}^{|m|}$ ($\mathbf {g}^\mu \in \{0,1\}^{|\mu |}$ respectively). As a shorthand notation, we will use the symbol $g$ to denote the tuple of the latter, i.e., $g=(\mathbf {g}^m, \mathbf {g}^\mu )$. Having these definitions on hand, we can define the problem to be solved in this paper.

#### Definition 1

(CircRNA annotation problem) For a circular RNA, let $\mathbf {A}^{m, \mu }$, $\mathbf {a}^{\mu , c}$ be its interaction graph. Decide whether the circRNA should be annotated with a term $g=(\mathbf {g}^m, \mathbf {g}^\mu )$.

### Proposed statistic

To solve the problem, we will develop a simple yet powerful statistic to annotate a circRNA. The concept is based on the ”guilt by association” principle [16, 21]. The circRNA should be annotated with a term if and only if this molecule frequently interacts with miRNAs (and through them indirectly with mRNAs) annotated with the term. We will capture this frequency in statistic *s*. This statistic will quantify the size of the neighborhood of the circRNA that is annotated with the term. As the complete tripartite graph is only a unification of two bipartite graphs and remains fixed for the circRNA, we might calculate this number precisely:

1 $$\begin{aligned} s(c, g) = (\mathbf {a}^{\mu , c})^T \mathbf {g}^\mu + (\mathbf {A}^{m, \mu }\mathbf {a}^{\mu , c})^T \mathbf {g}^m. \end{aligned}$$

The first addend shows how many paths of length one end in a miRNA annotated with the term. The term $\mathbf {A}^{m, \mu }\mathbf {a}^{\mu , c}$ shows how many paths go from $c$ to each mRNA. The second addend in Formula (1) calculates how many paths of length two terminate in an mRNA that is annotated with the term.

However, the frequency represented by $s$ is hard to explain without knowing the entire neighborhood. Larger neighborhoods, as well as more abundant gene terms, tend to generate a larger frequency. The importance of the term could better be captured by a relative frequency. Assume that we start a random walk in circRNA $c$. We might calculate the probability that this random walk ends in an RNA annotated with the term $g$. For a fixed circRNA, the size of the neighborhood is fixed. Therefore, the aforementioned probability is equal to﻿ $s(c, g)$ but for a normalization factor.

We will continue to use the number of paths represented in $s(c, g)$, knowing that they are only linearly scaled, preserving the ordering of the above-mentioned probabilities. To increase interpretability, we will further develop a normalized $s$ as well as the *p*-value for the statistic so that standard statistical reasoning is applicable. In this work, we will avoid random Monte-Carlo sampling (used among others in [6, 12–15]) and simulating the random walk and claim its low efficiency in our setting for the *p*-value calculation.

#### Normalization

As the value of statistic $s$ grows by definition with the size of annotation term and the size of the interaction graph (it gives the number of distinct paths to mRNAs and miRNAs annotated with the term), we present the user with a more explainable output. We normalize the statistic (1) by its expected value

2 $$\begin{aligned} \mathbb {E}\left( s(c, g) \right) = \frac{\Vert \mathbf {g}^\mu \Vert _1}{|\mu |} (\mathbf {a}^{\mu , c})^T\mathbf {1}+ \frac{\Vert \mathbf {g}^m \Vert _1}{|m|} (\mathbf {A}^{m, \mu }\mathbf {a}^{\mu , c})^T \mathbf {1} \end{aligned}$$

where $\mathbf {1}= (1, 1, \ldots , 1)^T$ is the vector of ones and $\Vert \cdot \Vert _1$ denotes the L1-norm. The expected value gives the expected number of random walks that end in an RNA annotated with the term if the annotations were assigned randomly. The user is then presented with the ratio of the statistic and its expected value

3 $$\begin{aligned} \frac{s(c, g)}{\mathbb {E}\left( s(c, g) \right) }. \end{aligned}$$

This ratio represents the normalized statistic. The value above 1 then stands for terms that tend to interact with the circRNA under observation more than expected as can be seen in Table 1.

#### Influence of individual RNAs

Once circGPA predicts that a circRNA should be annotated with a term, users might be interested in which miRNAs and mRNAs back up this annotation. In other words, knowledge of which RNAs connect the circRNA to the annotation term is important. Fortunately, it is possible to split $s(c, g)$ among individual molecules. A natural method of explaining how much the RNA adds to the statistic is to remove this RNA with all its incoming and outcoming edges from the graph. On such a modified graph, we recalculate the score and calculate the difference in the score value. We can calculate this difference for all miRNAs and mRNAs at once using linear algebra. We denote the vector of those differences $\Delta ^{m}$ ($\Delta ^{\mu }$) for mRNAs (miRNAs). For a vector $\mathbf {v}$, let $\mathop {{diag}}(\mathbf {v})$ denote a diagonal matrix with elements of $\mathbf {v}$ on its diagonal. Then we derive that

4 $$\begin{aligned} \Delta ^{\mu }&= \mathop {{diag}}(\mathbf {a}^{\mu , c}) \left( \mathbf {g}^\mu + (\mathbf {A}^{m, \mu })^T \mathbf {g}^m\right) , \end{aligned}$$

5 $$\begin{aligned} \Delta ^{m}&= \mathop {{diag}}(\mathbf {A}^{m, \mu }\mathbf {a}^{\mu , c}) \mathbf {g}^m. \end{aligned}$$

One can notice that the L1 norm of $\Delta ^{\mu }$ is equal to $s(c, g)$. We use values $\Delta ^{\mu }, \Delta ^{m}$ to sort mi/mRNAs in a report that shows the influence of individual RNAs. An example output will be seen in the "Results" section.

### *p*-value calculation

To understand and compare the values of statistic $s$ among different circRNAs and annotation terms, we need to calculate its *p*-value. The *p*-value cannot stem solely from *s* itself as other circular RNAs have a different number of connections to the remaining RNAs. In addition, more frequent annotation terms will reach higher scores. Formally, statistic $s(c,g)$ is an outcome of the statistical test, whose null hypothesis is that *the given *$c, g$ *pair is not related*. In other words, the null hypothesis is that circRNA $c$ has no preference in interactions with miRNAs (or mediated interactions with mRNAs) annotated with term $g$. The alternative hypothesis states that $c$ *should be annotated with* $g$ as $g$ is overrepresented in the neighborhood of $c$.

The *p*-value, in our case, represents the probability that a random annotation term of the same size in the same interaction graph reaches the same statistic $s$ or higher. The literal implementation of the null distribution simulation would thus be empirical random sampling with replacement [22]. In our case, this Monte-Carlo approach would be based on enumerating the random subsets of m/miRNAs of the same size as the evaluated annotation term and calculating the statistic value based on Formula (1).

This paper proposes an exact approach that does not depend on random trials but uses generating polynomials instead to compute the *p*-value. We should first reformulate the problem so that we can easily describe its mathematical solution. Denote $\Vert \mathbf {g}^\mu \Vert _1$ the number of miRNA molecules annotated by the term. Formally, $\Vert \mathbf {g}^\mu \Vert _1$ is the L1-norm of the $\mathbf {g}^\mu$ vector. Define $\Vert \mathbf {g}^m \Vert _1$ similarly. For each miRNA, there is a fixed number denoting its weight in the statistic (1). This weight is 1 if and only if the circRNA of interest is connected to the miRNA, zero otherwise. The weight is stored in the respective field of $\mathbf {a}^{\mu , c}$. Out of all miRNAs, we select $\Vert \mathbf {g}^\mu \Vert _1$. For mRNA, the weight can be seen in the respective field of $\mathbf {A}^{m, \mu }\mathbf {a}^{\mu , c}$. Out of all interacting mRNAs, $\Vert \mathbf {g}^m \Vert _1$ mRNAs are selected.

To calculate the *p*-value, the molecules of mRNA and miRNA are selected randomly given the weights and the fact that $\Vert \mathbf {g}^\mu \Vert _1$ and $\Vert \mathbf {g}^m \Vert _1$ need to be preserved. For now, we consider only miRNAs. Imagine a bag full of balls with numbers written on them. Each number is a field in $\mathbf {a}^{\mu , c}$ (one field equals one ball). Now we randomly select $\Vert \mathbf {g}^\mu \Vert _1$ balls from the bag and sum the numbers written on them. By repeating this procedure many times, we get the null distribution for the first part of the statistic (1). If we include a second bag with numbers taken from $\mathbf {A}^{m, \mu }\mathbf {a}^{\mu , c}$, we get the null distribution for the whole statistic.

Having built an informal intuition, we can proceed to introduce the generating polynomials by which we denote a polynomial which is a multiple of the well-established probability-generating functions [20]. Consider an mRNA that is connected by five paths to the circRNA. The weight of this mRNA is 5. In a random annotation term, this mRNA is either included or not. This gives two possibilities. We can formulate the generating polynomial for this mRNA as

6 $$\begin{aligned} 1 + x^5 y^1. \end{aligned}$$

The variable *x* keeps track of weights, *y* keeps track of the number of selected mRNAs. Having a simple graph with only one mRNA, we have two options for building a random mRNA set: either we use zero mRNAs, and the sum of weights is zero (the term 1, which equals $x^0 y^0$), or we use one, and the sum is 5 (the term $x^5 y^1$). If we also consider a new mRNA with weight 3, the resulting polynomial that represents the extended graph is

7 $$\begin{aligned} (1 + x^5 y^1) \cdot (1 + x^3 y^1) = 1 + x^3 y^1 + x^5 y^1 + x^8 y^2. \end{aligned}$$

We immediately see that if we select no mRNAs, we can only get the sum of weights 0; by selecting one, the weights will be either 3 or 5, and by selecting two, the sum of the weights will be eight. The coefficients by terms with $y^1$ show a single possibility of getting a weight of three or five. Another helpful view on the formula above might be as on a dynamic programming algorithm in a 2D array where the power of *x* denotes a row, the power of *y* denotes a column, and the coefficient is the number at the particular position of the table. Now, we can define the generating polynomial for a weight vector.

#### Definition 2

(Generating polynomial) Let $\mathbf {w}$ be a vector of weights (of mRNA or miRNA). Then the generating polynomial is

8 $$\begin{aligned} \hbox {genpoly}_{\mathbf {w}}(x,y) = \prod _{w\in \mathbf {w}} (1 + x^wy). \end{aligned}$$

Next, we define an operator that restricts the polynomial only on a selected power of one or more variables. We will denote the operator $\mid x^n$ and use it to denote only terms that contain $x^n$. For example, for the polynomial (7), operator $\mid y^1$ will return $x^3 + x^5$. The following theorem allows us to calculate the null distribution of the statistic (1).

#### Theorem 1

Consider statistic $s$ for a fixed circRNA $c$, interaction graph $\mathbf {a}^{\mu , c}$, $\mathbf {A}^{m, \mu }$ and annotation term sizes $\Vert \mathbf {g}^\mu \Vert _1$, $\Vert \mathbf {g}^m \Vert _1$. Then coefficients of the polynomial

9 $$\begin{aligned} \left( \hbox {genpoly}_{\mathbf {a}^{\mu , c}}(x,y) \mid y^{\Vert \mathbf {g}^\mu \Vert _1} \right) \left( \hbox {genpoly}_{\mathbf {A}^{m, \mu }\mathbf {a}^{\mu , c}}(x,y) \mid y^{\Vert \mathbf {g}^m \Vert _1} \right) \end{aligned}$$

are the null distribution of statistic $s$ up to a normalization factor.

#### Proof

From what precedes﻿, it can be seen that the first multiplicand coefficients are the number of ways to reach a particular value of the miRNA part of the statistic (1) by selecting a particular number of miRNAs. The restriction to the $y^{\Vert \mathbf {g}^\mu \Vert _1}$ ensures that the number of miRNAs in the annotation term is preserved. The same holds for the second multiplicand and mRNAs.

After multiplying the polynomials, the polynomial coefficients will hold the number of unique ways the value of the statistic can be achieved. The normalization to 1 then finishes the calculation of the null distribution. $\square$

Once the null distribution is calculated, the *p*-value is then obtained by a standard approach in which we sum probabilities of all statistic values greater than $s(c, g)$.

### Computational complexity

If we focus on the computational complexity of circGPA, most of the work is done in the Generating-Polynomial function. The two inner loops depend on variables *maxx* and *maxy*, where *maxx* is, in the worst case, linearly dependent on the L1-norm of vector $\mathbf {w}$; *maxy* is equal to the number of RNAs annotated with the terms. Their product, therefore, is linearly dependent on the product of $\Vert \mathbf {w}\Vert _1$ times the size of the annotation term. The two outer loops in function Generating-Polynomial do at most *n* operations for each unique non-zero entry in the vector of weights of count *n*. Sum of the fields of weight vector $\mathbf {w}$ is, therefore, the same as the number of evaluations of the two outer loops. The computational complexity of the two outer loops is in the worst case equal to $\Vert \mathbf {w}\Vert _1$. We may conclude that one call to the Generating-Polynomial function is in ${\mathcal {O}}(\Vert \mathbf {w}\Vert ^2 \cdot maxy)$. Other terms in function AnnotateCircRNA are asymptotically smaller than the runtime of the Generating-Polynomial function. The overall runtime is, therefore, in

10 $$\begin{aligned} {\mathcal {O}}\left( (\Vert \mathbf {a}^{\mu , c}\Vert _1)^2 \Vert \mathbf {g}^\mu \Vert _1 + (\Vert \mathbf {A}^{m, \mu }\mathbf {a}^{\mu , c}\Vert _1 )^2 \Vert \mathbf {g}^m\Vert _1\right) . \end{aligned}$$

### Referential approach

A standard approach for *p*-value calculation would be to enumerate subsets of miRNAs/mRNAs as random annotation terms. The size of the term is preserved, and we count how many times the score is higher than $s(c, g)$. This sampling Monte-Carlo approach then allows estimation of the *p*-value using the biased estimator $\frac{r+1}{n+1}$, where *r* is the number of trials with a high enough score and *n* is the number of all trials [23].

### Implementation

Using a naive implementation, we need at most $2^{|m|} \cdot 2^{|\mu |}$ multiplications to evaluate polynomial (9). This number is the theoretically possible maximum number of terms in polynomial (7). The real number is, however, much smaller. As polynomial exponents repeat, the bound can be tightened. The power of *x* goes from 0 to $\Vert \mathbf {a}^{\mu , c} \Vert _1$ in the case of miRNAs, and from 0 to $\Vert \mathbf {A}^{m, \mu }\mathbf {a}^{\mu , c} \Vert _1$ in the case of mRNAs. The *y* variable goes from 0 to $|\mu |$ (0 to $|m|$ in case of mRNA); however, relevant fields are only up to $\Vert \mathbf {g}^\mu \Vert _1$ ($\Vert \mathbf {g}^m \Vert _1$). The *x* variable can be trimmed similarly using the fact that only $\Vert \mathbf {g}^\mu \Vert _1$ (or $\Vert \mathbf {g}^m \Vert _1$) highest terms of $\mathbf {a}^{\mu , c}$ (or $\mathbf {A}^{m, \mu }\mathbf {a}^{\mu , c}$) may be used.

**Fig. 2:**
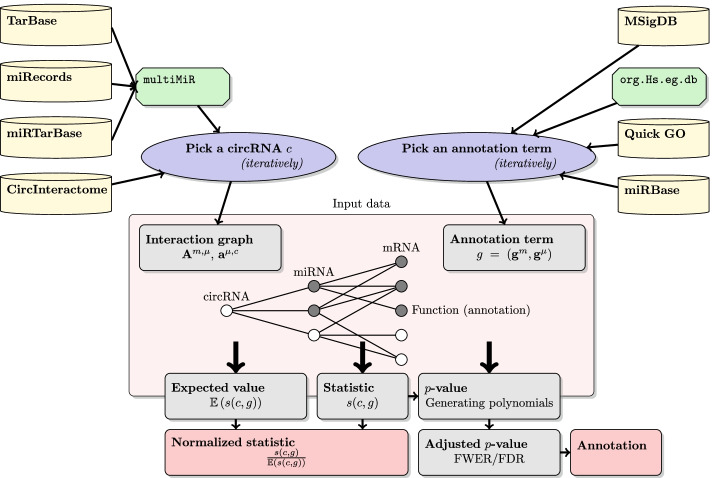
An illustration of the whole pipeline. The input graph is used for multiple annotation terms to calculate the statistic and its *p*-value. Later, the *p*-values are adjusted and used for annotation

If the weight of a mi/mRNA occurs more than once, we can exploit the binomial expansion instead of term-by-term multiplication in the Equation (8). The multiplication could be implemented using the dynamic programming approach and pointers. A similar approach was used for the fast *p*-value calculation of the unweighted GSEA [24]. Details can be seen in Algorithm 1. The whole pipeline is illustrated in Fig. 2.
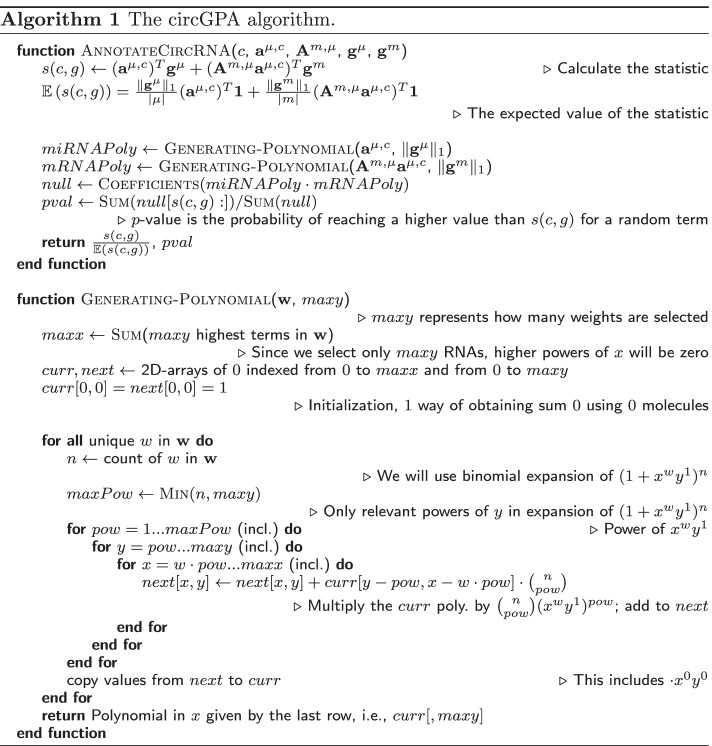

### Input data and used libraries

Our implementation combines R code and C++ code for the critical *p*-value calculation. To connect the C++ code and R code, we use the Rcpp package [25].

To construct the graph, we exploit several databases and R packages. The circRNA–miRNA interactions are downloaded from the CircInteractome [26] database that uses the TargetScan [27] interaction prediction algorithm. The miRNA–mRNA interactions are downloaded from the TarBase [28], miRecords [29], and miRTarBase [30] databases via the multiMiR package [31]. In the case of miRNA–mRNA interactions, we used verified interactions only.

The GO annotation for the miRNAs is downloaded using the miRBase [32] and ENA Quick GO [33] databases. Annotation of mRNAs is done via the org.Hs.eg.db R package. The annotation terms are obtained from the MSigDB database [34] C5 category using the msigdbr R package.

Other R packages used include miRBaseConverter, GO.db, biomaRt,stringr, httr, openxlsx, geometry, tictoc, ggnet, network and polynom.

The graph constructed in the presented way can annotate 3, 009 circRNAs. There are 1,761 miRNAs connected by 81, 391 edges. This means that one circRNA interacts with 29 miRNAs on average. One miRNA interacts with 50 circRNAs on average. The circRNA that interacts most with other molecules is hsa_circ_0000005 with 307 interactions. The miRNA with the most frequent interactions is hsa-miR-942, with 799.

The graph contains 19, 375 mRNAs with 465, 741 known interactions with miRNAs. Therefore, one mRNA interacts with 24 miRNAs on average, while one miRNA interacts with 264 mRNAs on average. The most frequent miRNA is hsa-miR-1-3p with 7491 interactions, the most frequent mRNA is NUFIP2 with 331 interactions.

We work with 10, 189 annotation terms. The average size of those is 82 mRNA or miRNA molecules. If we exclude annotation terms which are too narrow or too broad (see Sect. Results for details), we end up with 7075 annotation terms with 89 molecules on average. One RNA is annotated with 43 terms on average.

### All in one example

Consider the interaction network depicted in Fig. 1. In this figure, we have a circRNA of interest connected with 3 miRNAs that connect to 5 mRNAs. The edges in the graph can then be described by a vector and a matrix.

$$\begin{aligned} \mathbf {a}^{\mu , c}&= (1, 1, 1)^T,&\mathbf {A}^{m, \mu }&= \begin{pmatrix} 1 &{} 1 &{} 0 \\ 1 &{} 1 &{} 1 \\ 1 &{} 0 &{} 0 \\ 0 &{} 0 &{} 1 \\ 0 &{} 1 &{} 1 \end{pmatrix}. \end{aligned}$$

The annotation term contains 2 miRNAs and 3 mRNAs. It is formalized as

$$\begin{aligned} \mathbf {g}^\mu&= (1,1,0)^T,&\mathbf {g}^m&= (1,1,1,0,0)^T. \end{aligned}$$

The weights of the miRNAs and mRNAs are

$$\begin{aligned} \mathbf {a}^{\mu , c}&= (1,1,1)^T,&\mathbf {A}^{m, \mu }\mathbf {a}^{\mu , c}&= (2,3,1,1,2)^T. \end{aligned}$$

Which gives a statistic of

$$\begin{aligned} s(c, g) = 2 + 6 = 8. \end{aligned}$$

The first addend is for miRNAs, the second for mRNAs. The expected value of the statistic is

$$\begin{aligned} \mathbb {E}\left( s(c, g) \right) = \frac{2}{3} (1+1+1) + \frac{3}{5} (2+3+1+1+2) = 7.4. \end{aligned}$$

The normalized statistic is, therefore, $\frac{8}{7.4} \cong 1.08$. The generating polynomial for miRNAs is

$$\begin{aligned} (1 + x^1y)^3 = 1 + 3xy + 3x^2y^2 + x^3y^3. \end{aligned}$$

As we have two miRNAs that are annotated with the terms, we immediately see that there are three options for achieving two paths from the circRNA that end in an annotated miRNA (see term $3x^2y^2$ – variable *y* stands for the number of miRNAs, variable *x* stands for the number of paths). For mRNA, the generating polynomial is

$$\begin{aligned} (1 + x^1y)^2 (1 + x^2y)^2 (1 + x^3y) = \cdots + (2x^4 + 3x^5 + 4x^6 + x^7) y^3 + \cdots . \end{aligned}$$

**Fig. 3:**
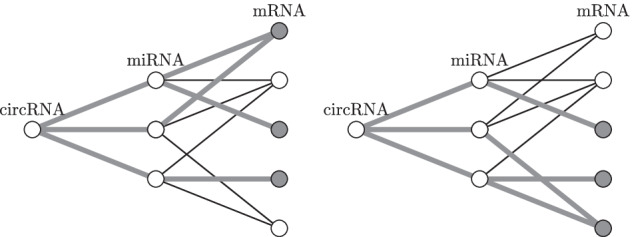
The generating polynomial of the mRNAs in this graph is equal to $2x^4 + 3x^5 + 4x^6 + x^7$. We see that there are two ways to annotate 3 mRNAs, so that there are 4 paths that end in an annotated mRNA. These two cases are illustrated in the figure, the paths are marked by bold gray

The relevant terms contain the variable *y* to the power of three – the term annotates three mRNAs. And the corresponding terms show that there are two options for selecting mRNAs such that there are 4 paths that go from the circRNA to an annotated mRNA. Those two options are illustrated in Fig. 3. Similarly, we can see that there are four options so that six paths end in an annotated mRNA and so on.

The generating polynomial for the whole statistic is, therefore,

$$\begin{aligned} 3x^2 (2x^4 + 3x^5 + 4x^6 + x^7) = 6x^6+ 9x^7 + 12 x^8 + 3 x^9. \end{aligned}$$

From the polynomial, we see that there are 12 ways to obtain a statistic value equal to 8. One of these is the situation depicted in Fig. 1 and solved in this example. The *p*-value is equal to the ratio of the number of combinations that reach a statistic value greater or equal to 8 to the number of all possible combinations. These can be seen from the polynomial or calculated as ${3 \atopwithdelims ()2}{5 \atopwithdelims ()3} = 30$. Hence, the *p*-value is $\frac{12+3}{6+9+12+3}$.

We can also see that the expected value is the same if calculated from the null distribution. There are six ways to get statistic equal to six, nine ways to get statistic equal to seven and so forth, i.e., the expected value is $\frac{6\cdot 6 + 9\cdot 7+ 12 \cdot 8 + 3\cdot 9}{6+9+12+3} = \frac{222}{30} = 7.4$.

## Results

We ran circGPA on a graph constructed on the human genome as explained in the "Implementation" section. For presentation purposes, we filtered the annotation terms based on their sizes. We excluded annotation terms which are too broad or too narrow. The narrow terms are difficult to evaluate statistically, the general terms suffer from low interestingness to domain experts. Reimand et al [35] argue that: ...*we often recommend excluding pathway GO terms with* $<10-15$ *genes and *$>200-500$ *genes, although upper bounds of *$200-2{,}000$ *genes can be found in the literature*. We decided to stick with the bounds researched by the authors and exclude by default terms smaller than 10 genes and larger than 1, 000 genes.

### Outputs of circGPA

For each circular RNA, circGPA generates a table with the statistic (1), the normalized statistic (as described in Sect. Normalization), and the *p*-values. Since we deal with many annotation terms in parallel, we have to adjust *p*-values for multiple comparison. We provide both FWER (Bonferroni [36]) and FDR (Holm [37]) adjusted *p*-values. The runtime of the *p*-value calculation is measured. An example output is shown in Table 1.

**Table 1:** A sample circGPA output

Set id	Size	$s(c, g)$	$\frac{s(c, g)}{\mathbb {E}\left( s(c, g) \right) }$	time (*s*)	*p*-value	Bonferroni	FDR
HP_SOFT_TISSUE_SARCOMA	115	38	3.61	0.04	1.40E-08	1.40E-04	1.39E-04
GOMF_MRNA_BINDING	287	64	2.44	0.16	2.78E-08	2.78E-04	1.39E-04
HP_GENITAL_NEOPLASM	142	41	3.16	0.05	5.78E-08	5.78E-04	1.93E-04
HP_SARCOMA	165	44	2.91	0.06	1.03E-07	1.03E-03	2.57E-04
HP_NEOPLASM_BY_HISTOLOGY	320	66	2.25	0.19	1.60E-07	1.60E-03	3.21E-04
HP_THIN_VERMILION_BORDER	329	66	2.19	0.20	3.48E-07	3.48E-03	5.80E-04
HP_THIN_UPPER_LIP_VERMILION	234	52	2.43	0.11	5.82E-07	5.82E-03	8.32E-04
GOMF_UBIQUITIN_LIKE_PROTEIN_LIGASE_BINDING	309	62	2.19	0.18	7.74E-07	7.75E-03	9.69E-04
HP_URINARY_TRACT_NEOPLASM	133	36	2.96	0.04	1.10E-06	1.10E-02	1.22E-03
GOBP_REGULATION_OF_MRNA_METABOLIC_PROCESS	334	64	2.09	0.22	1.75E-06	1.76E-02	1.63E-03

The table shows ten annotation terms with the smallest *p*-value that were obtained by annotating circRNA hsa_circ_0000228

**Fig. 4:**
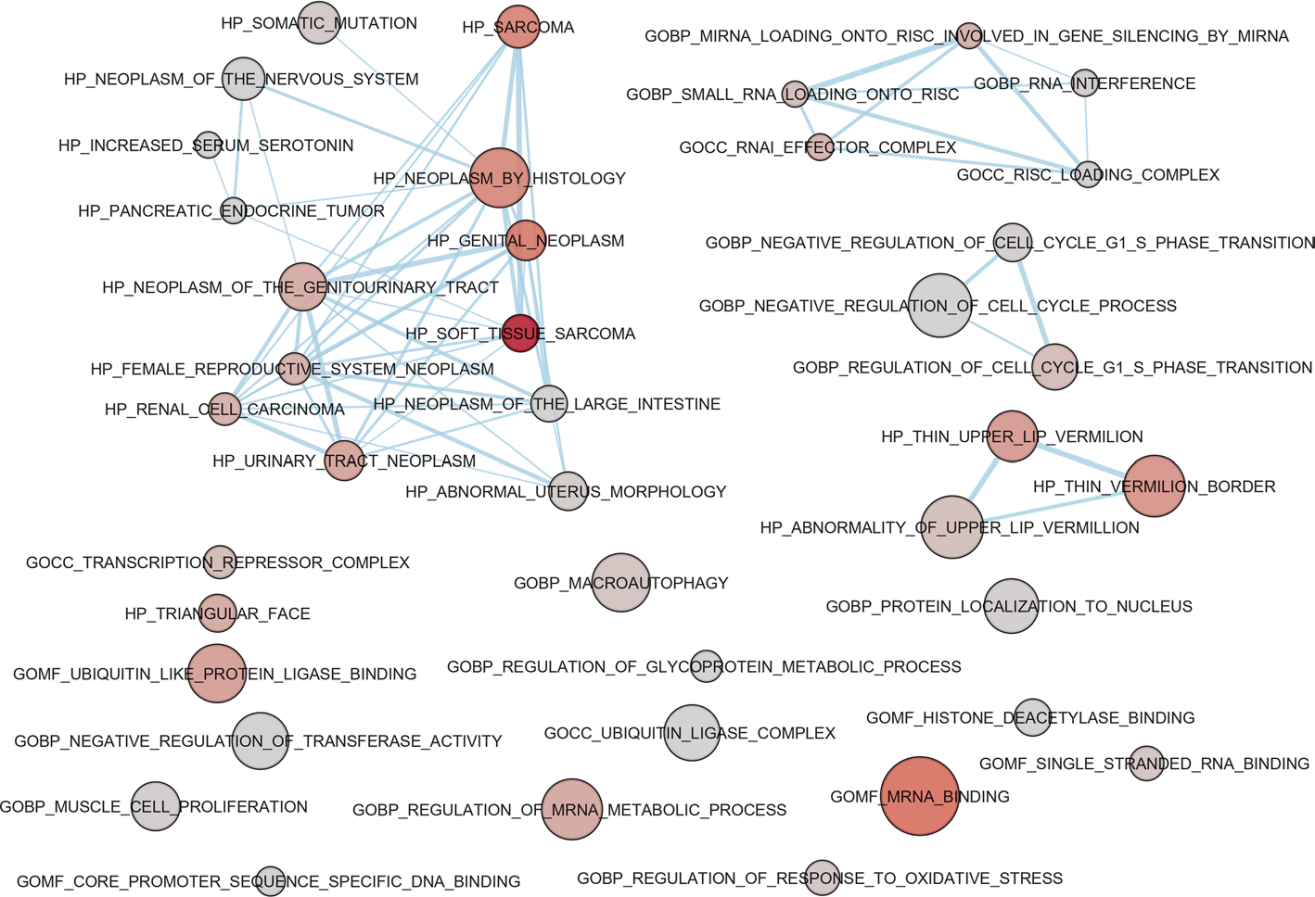
An example of a graph produced by the EnrichmentMap plugin of the Cytoscape program using the 40 most likely annotations of hsa_circ_0000228. The labels were moved manually so that they do not overlap. Several red circles that correspond to annotation terms with lower *p*-values can be noticed. Besides that, there are several clusters of terms that share genes. The biggest is located in the top left corner showing a set of terms connected with the reproductive and urinary systems. This indicates that the circular RNA might be connected with those systems. According to circBase [40], the sequence of hsa_circ_0000228 is located on the ZEB1 gene. According to the NCBI summary of publication [41], the ZEB1 gene shows the highest expression in the endometrium (out of 27 tissues presented) and is also highly expressed in the urinary bladder, placenta and prostatic tissues. Also, a recent publication has shown a connection between hsa_circ_0000228 and cervical cancer [42]. circGPA predicts a link of the circRNA to cancer as well, given the fact that HP_SOFT_TISSUE_SARCOMA is the term with the lowest *p*-value

**Fig. 5:**
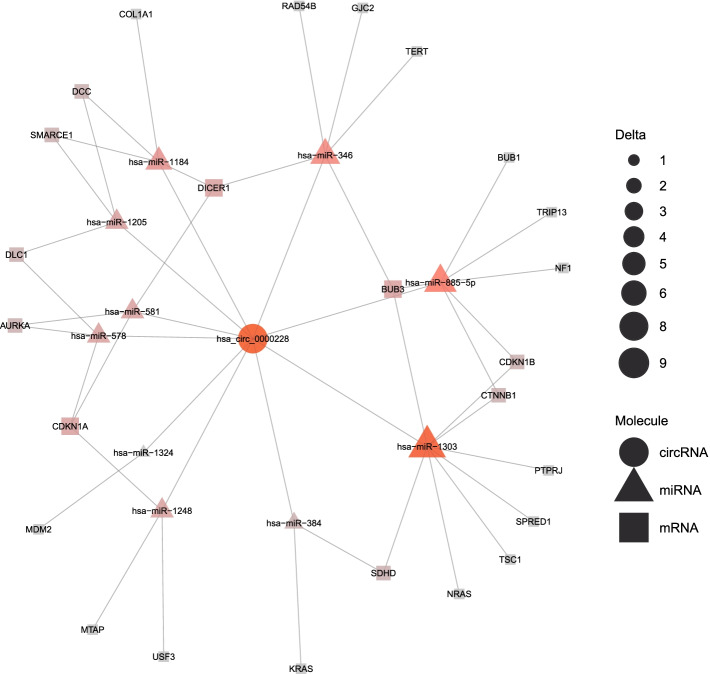
A network of miRNAs and mRNAs that back up annotation of hsa_circ_0000228 by the term with the lowest *p*-value - term HP_SOFT_TISSUE_SARCOMA. The size and color of each RNA shows how much the statistic (equal to 38) drops if the RNA is excluded from the graph together with all incident edges, i.e., the $\Delta ^{\mu }$ and $\Delta ^{m}$ values. See the "Influence of individual RNAs" section for details

For a visual presentation of the provided results, circGPA is able to generate an output in a form that can be processed by the EnrichmentMap plugin [38] of the Cytoscape program [39]. This tool visualizes multiple annotation terms found relevant for a single circRNA in a graph. The vertices correspond to the terms; their size corresponds to the number of genes in the term and the color is calculated from the *p*-value as in a heatmap. The edges are constructed so that their width represents the Jaccard index of the connected annotation terms – a wider line means a bigger overlap between the terms. An example of a produced output is presented in Fig. 4. Therefore, the graph for a circRNA shows predicted annotations in the context of the other annotation terms. As a result, the user is presented with information about the term-term overlap and clustering of the predicted annotation. Figure 5 shows an example of the circGPA report in which miRNAs and mRNAs back up annotation.

"To further test our method of *p*-value calculation, we implemented the usual sampling approach mentioned in the "*p*-value calculation" section. circGPA is deterministic and guarantees the exact *p*-values. The sampling approach is burdened with a random noise caused by the randomness in the selection of the subsets. Therefore, the *p*-values are not the same. At the very beginning, we thus configured the stochastic algorithm to approach the exact *p*-values. We worked with all the annotation terms relevant to hsa_circ_0000228. We found out that *p*-values closely match for $10^6$ and more random trials. We follow recommendations of [23] and estimated the *p*-values as $\frac{r+1}{n+1}$, where *r* is the number of positive Monte-Carlo trials out of $n = 10^6$. The Spearman’s $\rho$ for the two *p*-value vectors were equal to 0.99991. We also calculated the relative difference with a mean equal to 0.015 (i.e., on average, the *p*-values differ by less than $2\,\%$), standard deviation equal to 0.041, the maximum deviation equal to 0.82, and median equal to 0.006. If we eliminate the first 20 annotation terms with a *p*-value $1.6\cdot 10^{-5}$ and smaller, the mean of the relative *p*-value difference is 0.014, standard deviation 0.027, maximum deviation 0.372, and median 0.006.

### Runtime

**Fig. 6:**
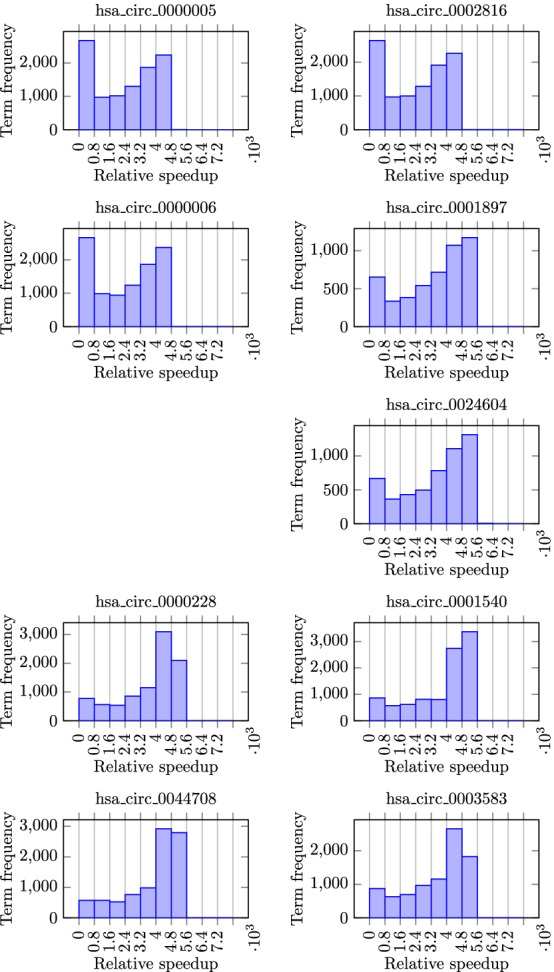
A histogram of the relative speedup of the *p*-value calculation using the generating polynomials compared to the sampling approach with the sample size $10^6$. The speedup is shown only for the case when the *p*-value is not equal to 1 (i.e., when $s(c, g) = 0$). CircRNA hsa_circ_0004624 is excluded from the plot as all *p*-values are equal to 1, meaning that the circRNA is not connected with any term

Then, we compared the circGPA runtime with the sampling approach using $10^6$ trials. The sampling was configured to allow for Monte-Carlo *p*-values to approach the exact deterministic ones calculated with generating polynomials. The comparison of runtimes is summarized in Fig. 6. In those experiments, we used the sample of circRNAs summarized in Table 2. It is obvious that circGPA overcomes the stochastic algorithm for all the tested annotation terms with speedups that vary from 0.16 to 48, 670. The average speedup per single *p*-value calculation proved to be 3, 150. If we compare the overall runtime requirements of 59 hours needed to calculate the *p*-values exactly on the testing circRNAs (see Table 2) and 1967 hours using the sampling approach, we can see that our approach is 33 times faster as a whole. Extrapolating this number means that our approach to calculating the *p*-values can save 65 years of CPU-time to annotate all circRNAs in our database (compared to approximately two years needed to calculate the *p*-values using the exact approach). However, the results of circGPA are worse on densely connected circRNAs that are over-represented in our dataset (see Table 2). The real measurements have shown that the circGPA requires 20 months to annotate all circRNAs in the database, including graph construction and disk access.

**Fig. 7:**
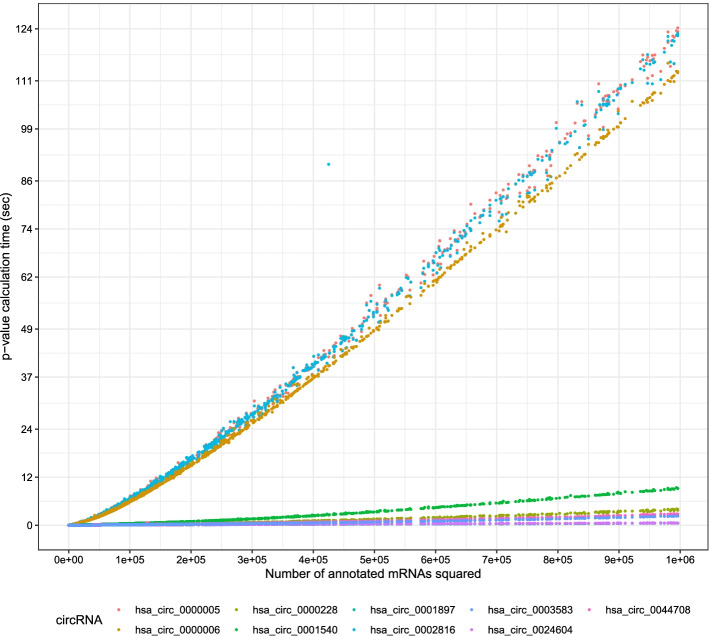
A plot of the time needed to calculate the *p*-value on $\Vert \mathbf {g}^m \Vert _1^2$. The plot was generated under the same conditions as Fig. 6

Further insights into the speedups provided by the algorithm are in Fig. 7, which demonstrates the runtime complexity of Algorithm 1. If we eliminate all terms that are constant for a given circular RNA from Formula (10) and re-evaluate the runtime of the two outer loops, the computational complexity is square of the size of the gene ontology term. As we see from Fig. 7, the runtime measurements are almost a line. The slope of the line is circRNA-dependent. For more connected circular RNAs, the slope is higher.

Figure 9 demonstrates that the decreasing number of trials in the sampling approach leads to a decrease in the accuracy of *p*-value estimation. It is impossible to straightforwardly decrease the number of trials and still approach the exact *p*-values. To reach runtimes observed in circGPA, the sampling algorithm would need to work with no more than 350 trials. However, the *p*-values then do not match the exact ones. We consider hsa_circ_0000228 with 352 sampling trials to illustrate the gap. The Spearman’s $\rho$ of the *p*-value vectors that capture those annotation terms that pass the 0.05 *p*-value significance threshold (the most likely to be truly relevant) is equal only to 0.93. The average relative difference between those is equal to 19, with a standard deviation of 178. The maximum relative difference is 2793, and the median 0.33. Excluding the first 20 terms with the lowest *p*-value decreases the maximum deviation to 176, the mean to 2.6, the standard deviation to 11.2, and the median stays 0.32. It needs to be said, however, that we followed the recommendation of [23] to use biased estimates.

### The *p*-values distribution

**Fig. 8:**
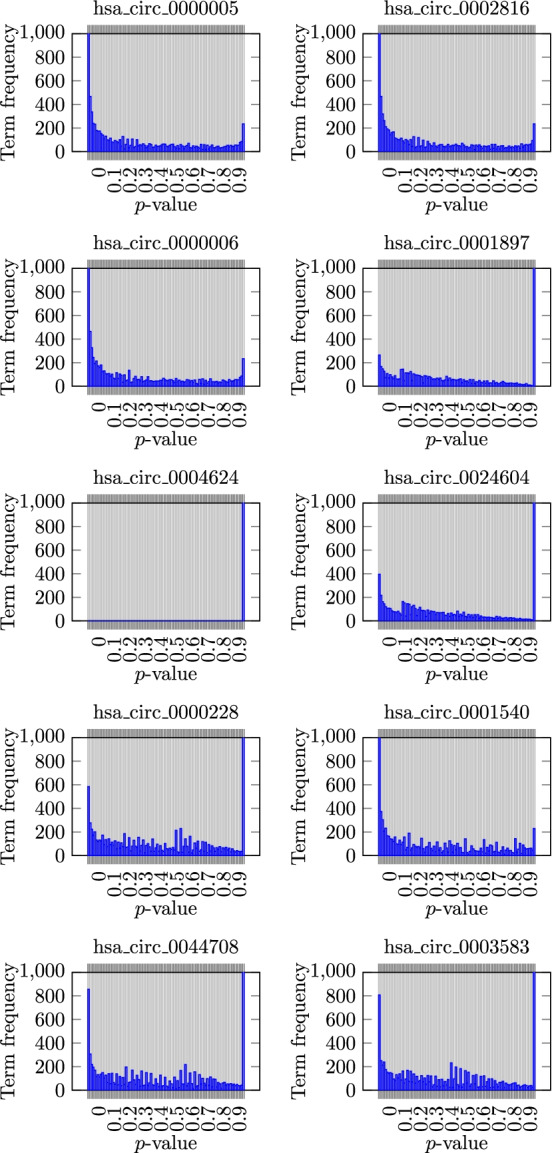
Histograms of the *p*-values before multiple hypothesis testing correction. The *y* axis is trimmed to 1, 000 annotation terms

The applicability of calculated *p*-values is demonstrated in Fig. 8. The histograms shown are mostly bimodal. The peak close to 0 *p*-values represents the cases where alternative hypotheses truly apply. The peak close to 1 *p*-value represents the cases where the network size and interactivity are not sufficient. Clearly, this peak is large, especially for the low-interacting circRNAs (see Table 2). We can notice that the most interacting circRNAs tend to attract more low-*p*-value annotation terms. On the contrary, for circRNAs with few interactions, there are only a few annotation terms with low *p*-values. This is actually the desired behavior as circRNAs that interact a lot may influence many other genes and pathways; however, circRNAs that have only a few interactions influence only a specific part of the cellular machinery. This situation is common in nature and can be explained by the well-known “80-20 rule” [43].

**Fig. 9:**
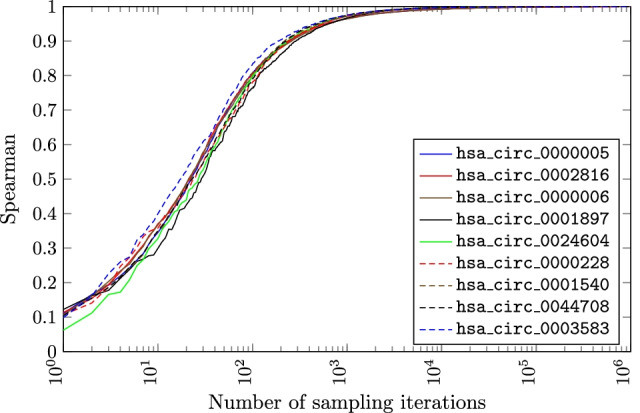
Dependence of Spearman’s correlation between the *p*-values calculated by Algorithm 1 and the sampling approach. The correlation is calculated for the annotation terms with *p*-value smaller than 0.05 (i.e., those that are likely to be checked manually). The plot excludes hsa_circ_0004624 as all *p*-values for this circRNA were equal to 1

**Table 2:** CircRNAs used in the experiments, along with the reason we included them

circRNA	Interacting miRNAs	Paths to mRNAs	Reason to include
hsa_circ_0000005	307	34043	Top-interacting
hsa_circ_0002816	305	34113	Top-interacting
hsa_circ_0000006	295	33470	Top-interacting
hsa_circ_0001897	2	302	Least-interacting
hsa_circ_0004624	2	0	Least-interacting
hsa_circ_0024604	2	325	Least-interacting
hsa_circ_0000228	26	1771	Used in development
hsa_circ_0001540	22	5037	Random choice
hsa_circ_0044708	11	1837	Random choice
hsa_circ_0003583	12	1234	Random choice

As the *p*-value calculation using the sampling approach takes up to two weeks on a single circRNA, we limited the experiments only to those circRNAs

## Discussion and related work

The previous section clearly shows that circGPA is an efficient tool for circRNA functional annotation. Let us compare it conceptually with similar existing tools. The closest tool is Cerina [10] which also employs the circRNA–miRNA–mRNA interaction network for circRNA functional annotation, including GO terms. Their stochastic approach is based on permuting the connections between a given circRNA and its interacting miRNAs/mRNAs. To reduce the size of the interaction network, Cerina binds the interaction and expression data and uses the Pareto-front-based algorithm for their integrative analysis. This step increases the efficiency of the stochastic algorithm and gives a chance to work with tissue-specific interactions only. We mention the possibilities of circGPA for integrative analysis in the "Conclusion" section.

The utilization of random-walk algorithms for functional annotation is wide. One of its early applications was the EnrichNet tool [14] for integrative analysis and gene annotation. Another usage is in [15], where the authors use random walks and circRNA similarity to predict circRNA-disease association. A similar approach utilized random walk for drug association prediction [44, 45]. The circRNA-disease association prediction problem was tackled using random walk with restarts [12, 46]. Fang et al. used random walks to predict miRNA-circRNA associations [13]. Close to the random walk with restarts algorithm is the PageRank algorithm [47] that has been developed for internet hyperlinks. If applied to the presented graph with circRNA–miRNA–mRNA interactions, both random walk with restarts and PageRank algorithms would lead to the same results as there is only a single source circRNA.

From the methodological point of view, the circRNA-disease association prediction is very similar to the problem solved by circGPA. The main condition for circGPA application in this task is the knowledge of miRNA/mRNA-disease annotations in the circRNA interaction network. These annotation databases exist. The miRNA-disease association databases include miR2Disease [48] with 3, 273 associations, and HMDDv3 [49], which contains 35, 547 manually collected miRNA-disease associations. Those associations were collected from 19, 280 scientific papers. For gene-disease associations, we can mention the DisGeNET database [50]. The accuracy of prediction could be verified against the known circRNA disease annotations. The CircR2Cancer [51] provides a list of 1, 439 manually curated entries. The Circ2Disease [52] is the most extensive database and contains 5, 368 manually curated entries. The CircR2Disease database [53] contains 725 associations.

One of the early papers from the circRNA-disease association prediction field is the Circ2Traits database [54]. The authors worked with two data sources to predict the associations. Firstly, it was a miRNA-circRNA interaction graph together with statistical tests. Secondly, the algorithm incorporates knowledge about single nucleotide polymorphisms. Recent tools for circRNA-disease association prediction usually take advantage of similarities between the circRNA pairs and disease pairs to predict the associations. For example, a tool named PWCDA developed in [55] constructs three graphs. The first graph represents circRNA similarity; the second graph represents disease similarity. Those two graphs are used to form a disease-circRNA association graph, out of which association scores are calculated. A similar approach was used in [56], where a Gaussian interacting profile is used to evaluate the similarities. There were frequent machine learning applications to circRNA-disease association prediction too. The work of Lei et al. [57] employs recommender systems to overcome the sparsity of validated annotations. The authors of [58] used convolutionary networks on the *k*-mer representation of the circRNA sequences. The disease similarities are captured using the disease ontology terms. The iGRLCDA tool [59] uses Gaussian interacting profiles, convolutional networks, and graph factorization. The authors of this paper developed a similar tool for drug-disease association prediction named HINGRL [60] too. The tool DWNCPCDA [61] uses DeepWalk. DWNCPCDA is based on the work by a similar set of authors - the NCPCDA tool [62] based on network consistency projection. This brief overview is far from complete. Other tools include, among others, [63–66].

A clear advantage of circGPA compared to the aforementioned circRNA-disease prediction algorithms is the existence of a *p*-value that allows filtering the predictions in a more advanced manner than selecting top *k* predictions. The runtime requirements of circGPA allow us to bulk annotate all known circRNAs with gene-ontology terms and provide the annotated results on https://ida.fel.cvut.cz/~rysavy/circgpa/.

## Conclusion

In this paper, we proposed an annotation algorithm circGPA that identifies prospective links between circRNAs and annotation terms. The algorithm is deterministic and based on generating polynomials. We show that this approach is both more effective and efficient than the alternative stochastic approach frequently applied in a similar context.

Our approach could easily be generalized for related tasks. Besides circRNAs, the long non-coding RNAs can act as miRNA sponges, and their annotation could be predicted too. As a whole, the approach is generalizable on any interactions which can be represented by a directed acyclic graph where leaves are annotated with binary concepts (annotation terms). Our goal is to decide upon the annotation of the roots of the graph (ncRNAs whose annotation is unknown). There are, however, computational limits to our approach. The *p*-value calculation is limited by the fact that we need to fit a potentially large table into the memory. The size of the table is the number of paths from the vertex of interest multiplied by the size of the annotation term.

When we compared circGPA with the sampling approach, we set the number of sampling trials as a constant. The only optimization we did was when the *p*-value was equal to 1. However, for high *p*-values annotation terms, it would be possible to stop the sampling earlier, knowing that the *p*-value will not be smaller than a threshold with a high-enough probability. Such approaches were proposed in [18, 19] and used in simctest R package. Similar ideas could apply to circGPA. We might use the generating polynomial to say that the *p*-value will not be smaller than a threshold without evaluating all polynomial coefficients. In the same manner, the weights in the loop of the Generation-Polynomial function could be sorted so that a bound on the *p*-value could be provided in the middle of computation.

In future work, we will look at the integrative analysis that deals with additional data modalities. So far, we have only examined interaction graphs. In the future, the annotation should stem from sequential data too in order not to rely on binary interaction records only. Also, tissue-specific expression data can help to minimize the impact of false-positive interactions with negligible expression and focus our analysis. The hierarchy of annotation terms could serve to regularize the eventual annotation records.

## Data Availability

The source code is publicly available on https://github.com/petrrysavy/circgpa-paper. The outputs of our code can be downloaded from https://ida.fel.cvut.cz/~rysavy/circgpa/.

## Footnotes

1 Additional graphical outputs took another 10 months of CPU time.

2 We define $\mathbf {a}^{\mu , c}$ as a binary vector, and $\mathbf {A}^{m, \mu }$ as a binary matrix. However, the approach can be easily generalized to the situation when $\mathbf {a}^{\mu , c}$ and $\mathbf {A}^{m, \mu }$ contain natural numbers which might capture, for example, the strength of the interactions or alternatively situations when a miRNA has two binding spots on a circRNA. The fields higher than 1 can be represented as parallel edges in the interaction graph which becomes a multigraph in this case.
